# Milking the data: Measuring milk off-take in extensive livestock systems. Experimental evidence from Niger

**DOI:** 10.1016/j.foodpol.2016.01.005

**Published:** 2016-02

**Authors:** Alberto Zezza, Giovanni Federighi, Amadou Adamou Kalilou, Pierre Hiernaux

**Affiliations:** a Development Data Group, The World Bank, United States; b Universita’ di Roma ‘Tor Vergata’, Italy; c ICRISAT, Niamey, Niger; d Géosciences Environnement, Toulouse, CNRS, France

**Keywords:** Livestock, Household surveys, Livelihoods, Questionnaire design, Milk, Niger

## Abstract

Milk is an important source of cash and nutrients for many households in developing countries. Yet, our understanding of the role of dairy production in livelihoods and nutritional outcomes is hindered by the lack of decent quality household survey data. Data on milk off-take for human consumption are difficult to collect in household surveys for a number of reasons which make accurate recall challenging for the respondent (continuous production and seasonality among others), introducing possibly severe biases in the computation of full household incomes and farm sales, as well as in the estimation of the contribution of livestock (specifically dairy) production to agricultural value added and the livelihoods of rural households.

This paper presents results from a validation exercise implemented in Niger, where alternative survey instruments based on recall methods were administered to randomly selected households, and compared to a 12-month system of physical monitoring and recording of milk production. The results of the exercise show that reasonably accurate estimates via recall methods are possible, and provide a clear ranking of questionnaire design options that can inform future survey operations.

## Highlights

• Collecting accurate milk off-take data is challenging in household surveys.
• Competing methods are tested in a randomized trial and compared to a benchmark.
• Results suggest some survey methods perform satisfactorily.
• Survey practitioners should consider these results when collecting recall milk data.

## Introduction and background

Despite the importance of the agricultural sector and its critical role in development policy and for poverty reduction, serious weaknesses in agricultural statistics persist throughout the developing world and are particularly pronounced in Africa. Of the 44 countries in Sub-Saharan Africa rated by the Food and Agriculture Organization, only two are considered to have high standards in data collection while standards in 21 countries remain low (Carletto, 2009).

Statistics on livestock stand out as an area in particular need for improvement. There are important technical reasons, besides institutional and political neglect, that explain why livestock data are particularly scarce or of dubious quality. Unlike crops, which are rooted in a specific tract of land and can be counted and measured, livestock are mobile, posing a challenge to enumeration even in sedentary livestock systems. The difficulties of collecting data on livestock are exacerbated by peculiarities in the management of livestock assets, in the mobility of some population groups that are especially reliant on livestock for their livelihoods (e.g. pastoralists), and by the fact that livestock products tend not to have one or two specific harvests at predetermined points in time, but tend to be produced either continuously or irregularly throughout the year, often with seasonal patterns.

The need of addressing the current shortcomings in the quality and availability of livestock statistics is only made more urgent by the rapidly increasing importance of the livestock sector. In developing countries as a whole, milk consumption almost doubled, meat consumption tripled, and egg consumption increased by a factor of five in the past fifty years, in what has been dubbed a ‘livestock revolution’ (Delgado et al., 1999). During the same period consumption of cereals increased only slightly and that of root and tubers actually declined (Gerosa and Skoet, 2013).

It is not uncommon for shares in excess of 60–70% of rural households in African countries to hold some livestock and depend on it to some extent for generating income or accessing nutrient dense foods (Davis et al., 2010). In rural Niger 3 out of 4 households keep some livestock according to recent national household level data (Bocoum et al., 2013), and according to FAO Statistics for 2010 (FAOSTAT, 2015) livestock contributes 28% of the net value of agricultural production in the African continent, with values substantially above average in several countries. In West Africa the share of livestock in agricultural production is consistently large, being 43% in Mali, 30% in Burkina Faso, and 38% in Niger, but other major countries also record sizeable shares, such as Ethiopia (35%).

Part of the neglect of livestock statistics materializes in underinvestment in actual data collection, but even when data are collected their quality is uncertain because of a lack of rigorous methodological work to assess the reliability of data collection practices. This paper aims to contribute to improving the practices for data collection on one specific item, milk, of major importance for livelihoods, income, food security and nutrition in many parts of Africa based on fieldwork in one of the African countries, Niger, where livestock constitutes the backbone of the rural economy.

Without reliable and timely livestock statistics it is hard to see how countries such as Niger can design, monitor and evaluate effective policies for promoting the role of livestock for poverty reduction and food security. The lack of high quality data on the dairy sector hinders both advocacy and policy analysis efforts aimed at informing actions to support livestock-based livelihoods. Household-level data and studies on the role of milk off-take for human nutrition and livelihoods are severely hampered by the difficulty of producing reliable estimates of milk off-take in small-scale livestock production systems.

Milk production offers an important source of cash income to many of the over 200 million poor livestock keepers estimated to reside in developing regions (Thornton et al., 2002, Pica-Ciamarra et al., 2015). For pastoral communities milk is often the sole source of calories and key nutrients, and a major source of cash income (Sadler et al., 2009). Some livestock products such as milk and eggs can help poorer households mitigate the effects of often large seasonal fluctuations in availability of cereals (Wilson et al., 2005). Hoddinott et al. (2015), using Ethiopian data, found empirical evidence to support the hypothesis that cow ownership in underdeveloped rural settings is a key driver of the milk consumption and linear growth of young children.

From a nutritional point of view, milk is a good source of dietary fat, energy, protein and other nutrients (Wijesinha-Bettoni and Burlingame, 2013) that brings “important nutritional benefits to large segments of the population of developing countries” (Muehlhoff et al., 2013: p. 5). In particular, milk can provide substantial amounts of nutrients such as calcium, magnesium, selenium, zinc, riboflavin, vitamin B12 and pantothenic acid (Weaver et al., 2013). Milk can help provide children of age 6–24 months that are not being breastfed adequate quantities of fat, which is crucial in their diets because it contains essential fatty acids, facilitates the absorption of fat soluble vitamins, and enhances dietary energy density and sensory qualities (Dewey, 2005).

Milk consumption has also been associated with secular growth in height whether in industrialized and developing countries (Japan, India) or in pastoral societies (Weaver et al., 2013, Hoppe et al., 2006). A review of the available evidence, laments that despite the observed increase in milk production and consumption world-wide, child undernutrition and micronutrient deficiencies that could be alleviated by increased intake of milk and other animal source foods remain highly prevalent. In developing countries, both milk and meat intake improve growth indicators, micronutrient status, and cognitive performance (Dror and Allen, 2011).

In general, it is hard to appreciate the role of milk and dairy production in household level livelihood studies in developing regions, because of the generally poor state of agricultural statistics in these countries, and because of the practical difficulties in measuring milk off-take in household surveys. Milk off-take is difficult to measure in household surveys because: (a) lactating females can be milked daily (often twice, mornings and evenings), but with seasonal patterns; (b) milk production varies depending on the lactation stage; (c) milk can be left in the udder to feed suckling youngs; (d) reproductive and lactating females may be present but not necessarily being milked. These potential sources of measurement error combined make the valuation of milk off-take particularly challenging in household surveys, introducing possibly severe biases in the computation of full household incomes and farm sales.

This paper presents results from a validation exercise implemented in Niger, where two alternative survey instruments were administered to randomly selected households, and then compared with the results of a physical monitoring of milk off-take over a 12-month period. The immediate objective of this work is to draw lessons for questionnaire design by selecting the best performing options and identifying outstanding issues. The ultimate goal is to contribute to a better understanding of the role of animal production in livelihoods and nutrition, which can facilitate more effective policy and program design.

The focus in the paper is on one specific family of household surveys, the Living Standard Measurement Study (LSMS). This is one prominent type of household survey widely implemented in developing countries to monitor and analyze poverty and livelihoods. While this is just one example of a multi-topic household survey for livelihood analysis, we maintain that the lesson for questionnaire design assessed with this exercise can be applied beyond LSMS surveys. The paper is organized as follows. The next section outlines the overall design of the validation exercise and the survey instruments being tested. This is followed by two sections presenting the data and the results. The concluding section discusses the implications of this work for future data collection, and elaborates on ongoing next steps in furthering this line of work.

## Testing alternative survey instruments

### The context: survey validation work in developing countries

In their primer on methods for testing and evaluating survey questions, Presser et al. (2004a, p. 109) note how “pretesting’s universally acknowledged importance has been honored more in the breach than in the practice”. Even in countries with well-funded statistical systems, pretests are often limited to a rehearsal of survey interviews, usually on a fairly limited number of cases, which are then qualitatively evaluated by the survey teams so as to draw lessons on questions that seemed to pose problems to interviewers or respondents. Sometimes, this is complemented by a quantitative analysis of response frequencies and other simple statistics from the data collected during a pilot survey.

In most cases there is little that is systematic about these tests, despite the existence of techniques geared toward assessing the performance of survey instruments (see e.g. those reviewed in Presser et al., 2004b; Iarossi, 2006), and very little documentation is provided to users of the data on the contents of such tests. The evaluation of what ‘works’ is mostly left to the judgment and experience of the survey team.

More and more, however, survey practitioners are paying attention to pre-tests as means toward improving data quality. Also, specific methods are being developed, tested and codified and increasingly applied in survey practice (Presser et al., 2004b). While the use of such methods, and their documentation, is more commonly found in OECD country surveys, their application is gaining grounds in low income countries, including in Africa.

Despite the fact that the quality of the data should be of interest to researchers as much as the quantity, it is surprising how little attention the formal validation of household survey data collection has received in the literature. Researchers’ preoccupation with data quality results mostly in efforts to design and supervise survey work as well as possible, but very infrequently are the results of such efforts formally tested. There are some notable exceptions however, and our study aims to contribute to this small but growing strand of methodological literature.

Most of the existing literature on survey experiments and survey validation refers to the measurement of household consumption. Beegle et al. (2012b) tested eight alternative methods of measuring household expenditure, comparing personal diary as the benchmark to other diary and recall formats. They found significant differences between resulting consumption measures, with the correlation between under-reporting and both illiteracy and urban households’ status being particularly evident. In addition, Gibson et al. (2014) used data from the same survey experiment in Tanzania to obtain evidence on the nature of measurement errors, concluding that, as expected, errors have a negative correlation with the true value of consumption.

In the context of household consumption, another issue that has been analyzed is the extent to which the length of the lists of consumption items affects estimates of household expenditures. In a study in El Salvador, Joliffe (2001) showed that a more detailed consumption list resulted in higher estimates of mean household expenditures (by around 30%). This finding has clear implications for the resulting poverty estimates.

The impact of the level of detail of the questionnaire on key indicators has also been investigated in the field of labor market statistics. Dillon et al. (2012) considered if this aspect, together with the type of respondent, can explain the existing widespread variation in measurement of child labor statistics. Their results confirmed that the use of shorter modules results in lower estimated incidence of child labor, and affects other important aspects of the analysis of labor markets.

Scott and Amenuvegbe (1990) conducted an experimental study on 135 households in Ghana. Each of them was interviewed 11 times at varying time intervals, asking to report expenditure on the 13 most frequently purchased items. In this study, each additional day of recall resulted in a 3% decline of the reported daily expenditure.

The choice of reference period is also likely to have considerable impact in several domains. Beegle et al. (2012a) tested for recall bias in agricultural data, submitting questionnaires with different time spans between harvest and interviews in three African countries. An assessment of whether and how modalities of data collection in agricultural production may affect results is also provided by Deininger et al. (2012). While the former study provides evidence that recall questions work well for a number of agricultural variables, the latter shows how for events with high frequency and strong seasonal components, diaries may outperform recall questions in terms of resulting data quality.

Using data from two microenterprise surveys in Sri Lanka, De Mel et al. (2009) find that firms under-reported revenues by about 30%, and that requesting them to maintain account books had significant impacts of on both the revenues and expenses they reported, but not on profits. More generally, they argue that questions on profits give truer measures than asking about revenues and expenses.

What this literature shows is how data collection methods matter as much as analytical tools and statistical techniques for the conclusions of a study. Yet, researchers are often ill equipped for judging the extent to which data quality can be affecting their results, whether using data collected by others or data collected as part of their own research, since the survey instruments employed rarely undergo this type of systematic validation. In particular, we are not aware of similar work done for livestock questionnaire design in the context of household surveys in low income countries, which is the reason that motivated a joint effort by FAO, ILRI, and the World Bank (as part of the LDIA and LSMS-ISA projects^1^) to start the survey validation work that is documented in this paper.

### Milk off-take recall methods

LSMS surveys have typically lumped the collection of data on livestock products in one table listing the different products on the rows and a set of standard questions, common to all products and based on a 12-month recall period, on the columns. The module usually asks a variation on two rather simple questions: (1) “Number of production months in the last 12 months”, and (2) “Average production (off-take) per month during production months”. Sometimes these questions are asked for milk as a homogeneous product, sometimes the product is broken down by livestock species (cattle, sheep, goat) or by dairy product (fresh or curd milk, cheese, butter).

Because of the peculiarities of milk production recalled earlier (such as continuous production, seasonality, varying lactating capacity of animals, including over time), simple recall questions are likely subject to large errors. This has led livestock researchers and livestock survey specialists to devise more complex strategies to collect more accurate milk off-take data as well as an expanding set of additional information useful to evaluate milk production systems.

Examples of these elaborate approaches include the 12-month method developed by CIRAD (Lesnoff et al., 2010), which relies on the monitoring/recording of off-take over extended periods of time, as well as on techniques which, while based on recall approaches, prompt the respondent more in depth about the milk off-take system hoping that this will help increase the accuracy of the responses. In developing new survey approaches to be integrated in LSMS-type surveys that include an expanded agricultural focus, these approaches are useful, but need to be adapted to conform to both the objective of the survey as well as to the survey operations. The only way to assess whether a change in approach results in an actual improvement in data quality is to validate the new method via fieldwork, ideally in an experimental setting, while reproducing as closely as possible real survey conditions.

The main goal of LSMS-type surveys is to generate information on household living standards and livelihoods, in this case jointly with information on the productivity, profitability and returns to different activities households may be engaged in. The LSMS survey logistics are organized with mobile teams, that normally reside in each enumeration area for 3–4 days, and need to complete the survey operations in that location in that given time. It is therefore beyond the scope of the LSMS, in terms of both objective and logistics, the possibility to collect milk off-take data over extensive time periods, or in a way that allows calculating the complex demographic parameters often required by livestock sector specialists (e.g. parturition, prolificacy and mortality rates). The objective of an LSMS needs to be more modest, and limited to collecting a reliable measure of milk off-take that can accurately portray the role that milk has in the overall household livelihood strategy.

At the same time, LSMS-type surveys aim to look at the heterogeneity across households, so methods that rely on the application of technical production factors from literature (e.g. average milk production or off-take per animal in a certain environment) to variables that may be easier to measure in a survey (such as number of animals milked by the household) may result in accurate ‘average’ estimates, but may artificially reduce the observed differences in milk off-take (both in physical and value terms) across households. For most of the analysis performed with LSMS data, the analysis of the dispersion of the distribution is often as or even more important than the analysis of the measures of central tendency (means, medians). Also, the number of lactating cows, ewes, does milked, the volume of milk extracted, the number of months milking is practiced for are all management decisions that vary across households and herders, for reasons that include but go beyond the milk production potential of the animal as expressed by technical parameters. For these reasons, competing data collection methods will need to be evaluated not only on the basis of their ability to yield an accurate point estimate of, say, mean milk off-take, but also on their ability to return a distribution of observations that resembles as much as possible the ‘true’ distribution.

In view of these considerations, in developing the Niger survey validation we looked at two methods that are often applied in livestock sector surveys, but also seemed to hold promise of being adaptable to both the questionnaire design and logistics of LSMS survey operations. In what follows we will refer to these two methods as the “Average Milk per Day” (AMD) and the “Lactation Curve” (LC) methods.

The two questionnaires are identical, except for one question on milk off-take. Both questionnaires are asked at the level of each animal species (cattle, sheep, goats, camels), and start off by prompting the respondents about the number of months during which animals were milked for human consumption, and how many animals were milked on average during each of those months. The questionnaires differ in that the AMD asks for the average quantity per day off-taken during the reference period,^2^ whereas the LC questionnaire asks about the amount of milk off-take on average from the animals milked at three, or four, different points in time: one week, one month, three and six months after parturition. The two modules then continue asking the same set of questions on issues of whether calves/lambs/kids were allowed to suckle, about the time duration between parturitions, and about the disposition of milk off-take (consumption and sales either fresh or after transformation into dairy products).

Annual household milk off-take can be calculated from both questionnaires. In the AMD this is done by simply multiplying the average daily off-take by 30 days (to get to monthly off-take per animal), then by the number of off-take months and by the number of animals milked. In the LC methods the calculation is somewhat more complex, as annual off-take is calculated as the area under each animal’s lactation curve, or rather milk off-take curve.

In all mammals lactation starts shortly after parturition, a peak in lactation is reached early in the lactation period, followed by a gradual decline to the end of the lactation. The timing of these periods, and the overall length of the lactation vary by animal species, and by breeds, and with climatic, grazing, watering and several other factors. Besides that, what the survey asks is not lactation as such, but the amount of milk that is taken off for human consumption, which is a decision variable for the farmer.

**Fig. 1:**
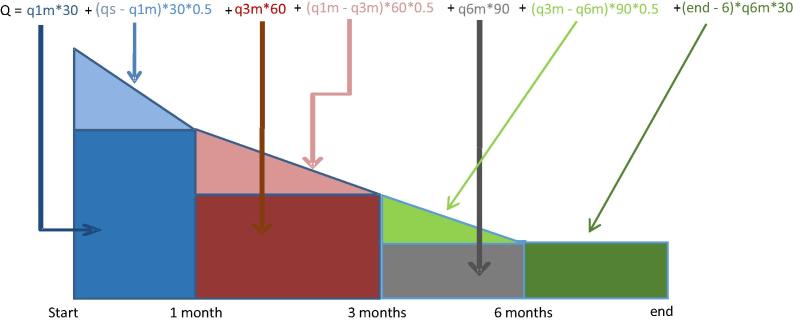
Computing milk off-take using the LC method.

Total milk off-take can therefore be approximated, assuming a constant value of off-take between the last point in time for which the recall asks and that of the end of the milking period,^3^ as the area under a curve such as depicted in Fig. 1. In the most general case of four monitoring points, the corresponding formula can be written as:

Q=q1m∗30+(qs-q1m)∗30∗0.5+q3m∗60+(q1m-q3m)∗60∗0.5+q6m∗90+(q3m-q6m)∗90∗0.5+q6m∗(end-6)∗30

where *Q* is the total milk off-take per animal in one lactation, *qs* is the average daily quantity of milk off-taken per animal at the start of the lactating period (one week after parturition in the Niger LC module), *q*1*m, q*3*m* and *q*6*m* are respectively the off-take one month, three and six months after parturition, and *end* is the average number of months of milk off-take per animal. For animals with shorter lactation periods such as ewes and does, more parturitions (and hence lactations) may fall within the 12 months of the survey reference period. In such cases, the presence of a question on the average interval between parturitions allows attributing a quota of the second lactation to the survey reference period (Njuki et al., 2011). In this paper we focus on cattle milk over a 12 months reference period, which rules out the possibility of multiple lactations for any one animal as the calving interval for cattle is longer than 12 months.

With the LC method respondents are asked to recall more information (milk off-take at different stages of lactation) but to only average out this information across the animals they have milked. In the AMD method, respondents are required to report only one figure, but to obtain that via an implicit process of averaging not only across animals but also across lactation stages. What process is easier for the respondent and more likely to return an estimate closer to the ‘true’ value is an empirical question, and the main question this paper aims to address. Whether it is easier for respondents to answer questions about an average animal or about the entire herd is also an empirical question.^4^ In the study area each animal is milked separately but the milk extracted is poured in a single pot (or a series of pots), thus the herder in charge of milking may have a feeling for both the average volume of milk from a cow and the average volume of milk collected from the herd. After some piloting in the field, it was felt that respondents found it easier to report about off-take per herd, as the milk is collected for all animals into one container, once or twice a day.

It should be noted that in empirical applications, particularly in specialized dairy livestock surveys, the LC methods is often implemented with respect to one or more specific animal(s) selected at random from the respondent’s herd. This also allows capturing the possible variation in the lactation stage of different animals throughout the year, where this is a concern. In living standard surveys, where livestock is just one component of a more complex survey, this would not however be practical. Interviews are often carried out at the household residence, and the herd may not be physically present for the selection of the animals to be made. Also, if both large and small ruminants need to be enumerated that would require identifying different animals in potentially different locations at the time of the interview. The time and cost implications of this for a large scale national survey are likely to be prohibitive. The results presented below therefore need to be interpreted with the qualification that they apply to the lactation curve method as applied in this exercise. Particularly when there is variability across animals and over time, respondents may find it easier to recall milk off-take at specific points of the lactation for specific animals as opposed to an hypothetical ‘average animal’ as implied by the way the method was applied in the Niger survey we report about.

In the study area milk off-take has a markedly seasonal pattern, and animals tend to follow similar patterns over the year so that one does not expect to find significant differences in the lactation stage across animals at any point in time. The seasonal increase in number of cows milked and the increase in volume of milk off-take by cow, both contribute to the increase in milk off-take by farm during the wet season (August to December), then progressively decreasing reaching the minimum off-take level from March to June. Variability in the lactation stage across animals is therefore not a major concern in the area the data collection for this study took place.

**Fig. 2:**
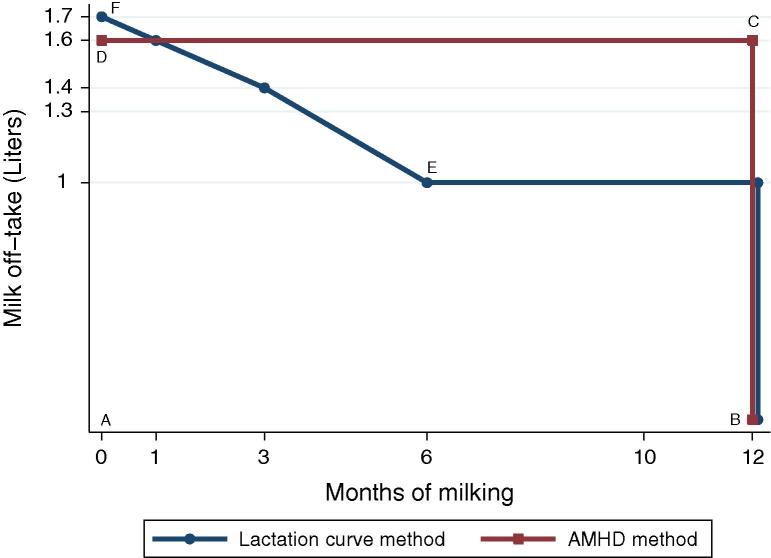
Comparison of recall methods.

Some livestock survey practitioners suggest that the response given to the AMD question may result in an overestimate of the quantity of milk collected as the response patterns may lead to estimating the area under a rectangle that will largely be above the lactation curve triangle. Fig. 2 illustrates the point, using hypothetical values not too dissimilar from the data in our Niger cattle milk off-take study. In calculating total milk off-take from the AMD method one is essentially computing the area of the rectangle ABCD, where AB is the number of months milk was collected and BC is the monthly quantity (in liters) of collected milk.^5^ Suppose the true shape of the off-take curve for the respondent was equal to the line BEF, and it becomes evident how AMD would result in an overestimate of milk off-take.

The AMD method can be administered for different recall periods, as it is often argued that shorter recall can improve data quality. This is especially true for variables that are characterized by seasonal patterns, which is the case for milk off-take. In the case of the LC method this is not feasible as a, say, 6-month recall period would likely be shorter than the lactation/off-take period, thus complicating the task of the respondent as some of the points in the off-take curve may fall outside of the recall period.

It is also often found that additional questions related to the main object of the recall can be useful in aiding the recall by the respondent. For that reason, in the exercise described in this paper, we also experimented in combining the AMD with the LC questions. The idea is that if a respondent may provide a more accurate answer when asked to estimate average off-take if she is also invited to recall average off-take at different stages over the lactating period, than if asked to provide that figure directly.

In the exercise reported on here we compare the following methods: (a) the LC method; (b) the AMHD method with a 12-month recall; (c) the AMHD method with a 12-month recall and linked to the LC method questions; (d) the AMHD method with a 6-month recall. All are compared against a benchmark constructed by the physical monitoring of daily milk off-take measured every fortnight over a 12-month period. We also provide some evidence on the performance of the AMAD variant of the AMD method. Before discussing the results of these comparisons, we now turn to a description of the data.

## Data

The main data set analyzed in this paper comes from fieldwork that took place in the Dantiandou district in Niger, between April 2012 and June 2013, and is referred to here as the Dantlait survey. The fieldwork was managed by two experienced enumerators, and a supervisor. The team monitored the milk off-take of 300 households over 12 months, as well as associated livestock management, together with household consumption and sale of dairy products. The team also administered 6-month recall questionnaires on 200 households, and 12-month recall questionnaires to 400 households.

The first 200 household farms were randomly sampled among the 835 household farms documented in 2009 and 2010 for the Livestock Climate and Society (ECliS) project.^6^ These 835 households live in 13 villages and associated camps within the district (commune and canton) of Dantiandou (80 km East of the capital Niamey). A large data base is available on the composition of each household, its economic activities (including cropping, breeding livestock, forestry, and off farm), the composition and number of livestock, milking practice, consumption and sale of dairy products. This data base was used to stratify the households based on the type of dwelling (either village or camp), which largely matches with ethnic affiliation (Zarma/Fulani), and on the size of the cattle herd (less than 5, 5–15, more than 15 adult females). The additional 100 households were selected in 13 additional villages from the district of Dantiandou (5340 households and 45 villages in total), based on the 2008 national census.

The monitoring method targeted the assessment of the daily milk off-take in each of the 300 sampled households. For each sampled herd, the milk off-take was measured one day every fortnight adding morning and evening milking when applicable. At each milking, the total milk off-take of the herd was poured in a transparent plastic pot devoted to that measure. The level reached by the milk was marked on the outside of the pot with a marker by the herder. To assess milk volume, the research assistant weighted the plastic pots empty and when filled with water up to the mark done on the side of the pot. The pot weights were recorded on the herd recording form together with the number of lactating females, and the number of lactating females milked re-actualized at each visit. Equipped with a motorbike, each of the two enumerators monitored about ten farms per day (one or two visits depending on milking practices), with revisits every two weeks. The objective of the physical monitoring was to construct a measure that could be used as a benchmark against which to compare the different recall methods.

Camp households have larger cattle herds on average than village households (7.2 vs 4.4). The mean number of lactating cows in the course of the year is 3.4 vs 1.8. Only a fraction of the lactating females are actually milked, on average 1.9 vs 1.3. Resulting mean milk off-take is low, at 2.1 liters per day in camps and 1.3 liters per day in village farms. There are large seasonal variations in milk off-take, the wet season and first part of the dry season (‘cool’ season) contrasting with the late dry season (‘hot’ season), by a factor 2 in camp farms and factor 1.5 in village farms. These seasonal variations are explained by the reproductive cycle of the cows (peak of births in early wet season), the better quality of grazing resources, but these reasons are modulated by the herder’s decision (i.e. share of lactating cows actually milked, milking in the morning/evening or both, volume of off-take). It appears for example that the volume milked (0.8–0.9 liters per cow and per milking) does not vary with farm type, morning or evening milking, and position along the lactating curve. Sparing milk for the calves drives the practice of milk off-take especially in camp farms.

Recall questionnaires were asked to 200 farms (141 of which had also been monitored) in December 2012, and to 400 farms (269 of which had also been monitored) in May–June 2013. The December survey included a 6-month recall AMHD questionnaire. The 400 households interviewed for the 2013 survey were randomly split into two groups, with one being administered a 12-month AMHD recall, and the other a LC module, where the LC questions were followed by an AMHD question. We are therefore able to compute recall measures based on the four measures described above (6-month recall, which we also annualize by multiplying it by 2), LC curve, 12-month AMHD, and 12-month AMHD combined with LC as a recall aid for the respondent.

Of importance to the design of the study, we observe no significant differences between the two groups in which our sample was randomly split. That provides confidence in that the random design on which the survey is based worked, and that the compared groups show no systematic difference other than the fact that they have been asked different questions. Table 1 summarizes the descriptive statistics for the key groups in which the sample has been split for fieldwork and analysis. Only one variable (months of milking) shows statistically significant differences between the LC and the AMD questionnaires. However, a further separation of those who responded to the LC or to the AMD questionnaires into the physically monitored and not monitored groups, does not show any statistically significant differences. Most of the comparisons we base our conclusions on refer to the 269 monitored households only, so that even if there was a bias in the selection of households to monitor it would not affect the comparisons. The non-monitored households were mainly added to the sample to obtain more statistical power in the comparison of means.

## Results

Our initial expectation was that the LC method could provide an improvement of milk off-take estimates over the AMD method, which we expected to overestimate off-take. The key results from the validation exercise are reported in Table 2.

The first result is that the AMD recall methods perform much better than expected, and appear to be superior to the LC methods. The deviation of the median values from the median of the milk monitoring is surprisingly close to the value obtained via the physical monitoring with a difference of just 21 l (about 3%). The deviations for the mean values are somewhat larger but still acceptable at 30 l (3% of the monitoring value for the 6-month recall, up to 6% for the other variants).

Secondly, for the LC method the results are less satisfactory. Deviations from the ‘gold standard’ represented by the physical monitoring range between ‘acceptable’ levels at 6% and 10%, when median values are considered (for the 4- and 3-point measures, respectively). If one considers deviations from the average value of the monitoring, however, differences increase to 13% for the 4-point LC method and 37% for the 3-point LC method. In general, the 4-point method appears to perform significantly better, thus justifying the extra answer required of the respondent.

Thirdly, the results show that a major feature common to both the AMD and LC methods is that they over-estimate the dispersion around the mean (as measured by the standard deviation), and particularly so for the LC method. Among the AMD variants, the highest standard deviation is 1.4 times the standard deviation of the monitoring. For the LC method the ratio is 1.8.

Within the AMD methods, shortening the recall period to 6 months appears to perform as well as the 12-month recall, without major improvement in accuracy. In this particular sample the 6-month recall did not generate any extreme value, which happened for the 12-month survey, but it is hard to generalize this result, as it is linked to the answers of a few respondents.^7^

Another interesting result is that the AMD method, when integrated with the LC questions, returned substantially more accurate results than when the LC questions were not included. It appears that introducing LC as a recall aid helped respondents to average out to values closer to the ‘true’ value of the monitoring. This is particularly true for camp households, which are characterized by both higher off-take values, and higher seasonal fluctuations. Yet, these conclusions are based on only few observations in the left-hand tail of the distribution curve.

**Fig. 3:**
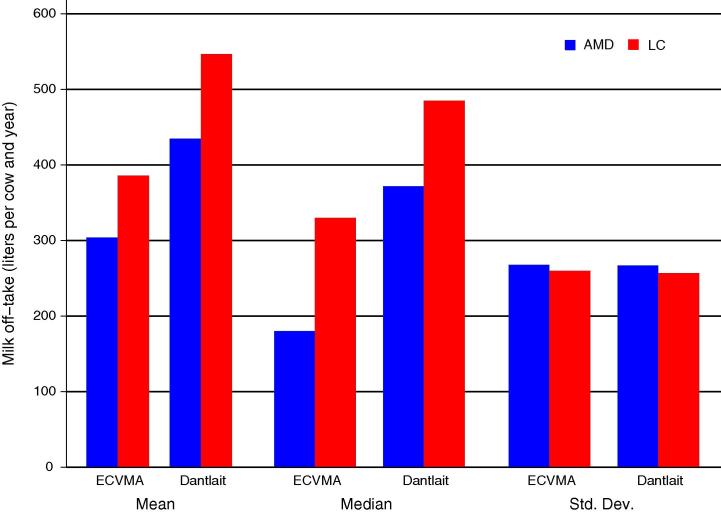
Comparison of mean, median and standard deviation measures of milk off-take estimates (liters per cow and year) from AMD and LC methods in Dantlait and ECVMA surveys.

*Source:* Dantlait and ECVMA surveys.

Earlier in the project, a LC questionnaire and a 12-month AMAD recall had been included in the Enquête Nationale sur les Conditions de Vie des Ménages et l’Agriculture (ECVMA) survey implemented in 2011 by the Institut National de la Statistique (INS) of Niger, on a nationally representative sample of 3968 households. While it was not possible to construct a monitoring benchmark for this large nationally representative survey, the results of the comparison between the two recall methods can be interpreted in the light of the conclusions emerging from the Dantiandou survey and monitoring. Fig. 3 reports the mean, median and standard deviation measures of milk off-take per cow in both data sets. The patterns, in terms of differences between the LC (based on 3 points) and AMD methods, are very similar in the two surveys. This supports the idea that the Dantlait survey results can be extrapolated to a sample of households in other parts of Niger, conducted as part of a larger scale, national survey operation conducted by the national statistical office.^8^

It is important to note, however, that the Dantlait survey was limited to cattle. Small ruminants have shorter lactating periods and the results may not apply to them. In the ECVMA data, for instance, milk off-take from ewes and does^9^ is substantially higher when estimated with the AMD method compared to the 3-point lactation curve method, which is the opposite of what happens for cattle in the same sample. Unfortunately, as discussed earlier, the ECVMA did not include a benchmark that allows assessing the accuracy of these estimates.

**Fig. 4:**
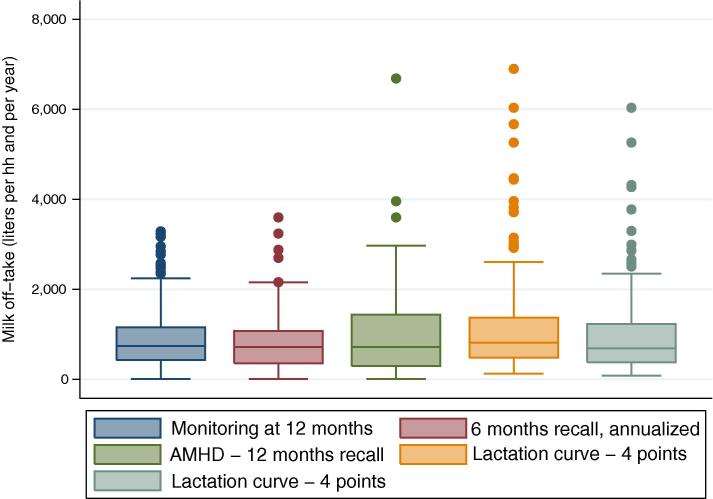
Box plots of household daily milk off-take (liters): monitoring and recall.

*Source:* Dantlait survey.

Besides providing reasonable average estimates, LSMS-type surveys are geared toward depicting the heterogeneity in households’ livelihoods and productivity. To that end, looking at how different indicators perform along the entire distribution, and understanding how well they can estimate the position of each household along the distribution is as, if not more, important as obtaining an accurate central tendency measure. For these reasons it is worth analyzing also the correlation and regression coefficients between the different recall methods and the monitoring benchmark (Table 3), and the box plots for the different measures (Fig. 4).

Looking at Table 3, it is comforting to observe that the implicit ranking of the different recall methods observed for the central tendencies (Table 2) is also confirmed by the overall correlation between the measures resulting from different recall methods. The annualized 6-month (AMD, top row) and the straight 6-month recall (bottom row)^10^ display the highest coefficients and *R*^2^, followed by the other 12-month recall methods in the order in which they appear in the table, and again pointing to a better performance of the 4-point compared to the 3-point LC variant.

The box-plots (Fig. 4) further support these results. To improve readability we only show five indicators, the monitoring, 6-month recall annualized, 12-month recall, and 3- and 4-point lactation curve. As shown in Table 2, Table 3, the 6-month recall method shows a little more dispersion than the monitoring, but in terms of median and overall distribution the fit is very good. The dispersion at the top end of the distribution increases with the less precise AMD methods, but remains broadly acceptable (even though it is to some extent subjective to define ‘acceptable’ in this case), and becomes substantially higher for both variants of the lactation curve method.

**Fig. 5:**
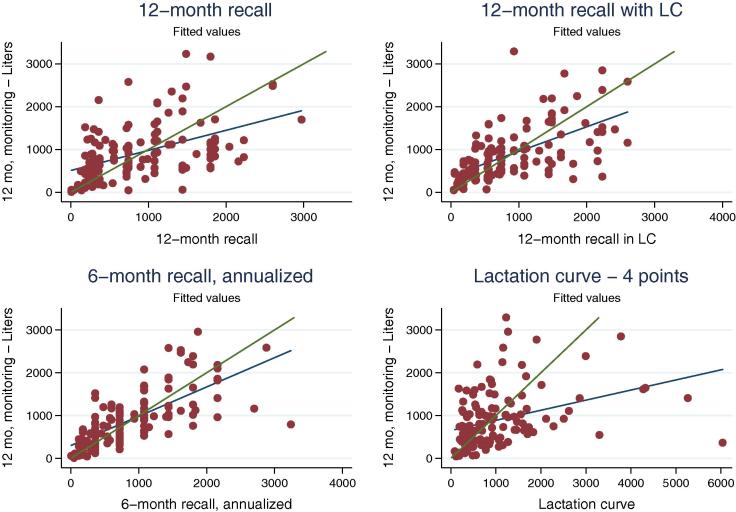
Household milk off-take (liters per year): scatter plots of recall against monitoring method.

*Source:* Dantlait survey.

We also plotted scatter plots of the different recall measures against the result of the milk monitoring. Results are reported in Fig. 5, where the solid green line represents the line of equality between the two measures, whereas the dashed blue line is based on a linear fit.

It appears that the methods that perform better when judged on the synthetic measures analyzed so far, also perform better with respect to individual household observations. The cloud is a lot more scattered in the case of the LC method than it is for the 6-month recall or the 12-month recall with the LC aid. Second, a fair amount of measurement error remains.^11^ More importantly, at this visual inspection the error does not seem to be randomly distributed, but tends to be negatively correlated to ‘actual’ (i.e. monitored) milk off-take. Respondents are more likely to under-report milk off-take if they produce larger quantities of milk, and they are more likely to over-report off-take when they produce smaller quantities. This is clearly a matter of concern for the analyst, as measures of income from milk off-take and productivity based on such data would be biased on ways that are correlated with other variables of interest.

For that reason, it is important to understand what are the correlates and determinants of the observed measurement error. Table 4 presents the results of a series of linear regressions where the percentage difference between the recall methods and the monitored milk quantities (the dependent variables, one for each method) are regressed against a set of covariates which we expect to influence the quality of the recall. The independent variables in the regressions include herd and production system characteristics, as well as other household and respondent features. Since we expect respondents to be less accurate in averaging out off-take over 12 months the higher the day-to-day variability in off-take levels, the first variable in the regression is the household-specific coefficient of variation of the monitored off-take, computed as the standard deviation of total milk produced for all cows divided by its mean. We also include variables that reflect differences in management or milking practices that may be systematically related to recall quality: whether the household is in a village or camp, whether cows are milked once or twice per day, the number of cows milked, and the duration of the last lactation period. A variable measuring the number of cows that receive feed supplements is included, as this indicator can be related to both milk off-take per cow as well as managerial ability or availability of resources on the part of the herder.

We hypothesize that respondents who are not exclusively focused on cattle rearing might recall events about this species less accurately, and we use information on ownership of other animals, engagement in activities other than agriculture and source of income from migration as additional controls. The number of mobile phones owned by the household is included as a proxy for overall wealth, as well as ability to access and process information, while age of the household head is included on the grounds that ability to recall may decline with age. On the other hand, if younger farmers are less experienced, response accuracy could actually increase with age. Since two different enumerators collected data in the field, we also include a dummy to control for possible differences in enumerator’s ability.

The most consistent, robust message that comes from this analysis is that the measurement error is correlated with the number of animals milked. The coefficient is of the expected positive sign, large in magnitude and statistically highly significant in five of the seven regressions we estimated. Interestingly, the coefficient is not significant only in the two methods based on the 6-month recall, suggesting that shortening the recall period may be an effective means to not only improve accuracy but also reduce bias.

Respondents living in the camps appear to be better able to recall the amount of milk off-take, and this is reflected in smaller measurement error. This may be linked to management practices, to the fact that livestock might be relatively more important in camps, and to reasons of ethnicity (Fulani herders are more likely to be residing in camps, compared to Zarma). It is hard to disentangle these effects and it should also be acknowledged that for reasons of collinearity, one should interpret with caution the regression coefficients that relate to management practices. This is the case for instance for the positive sign on the coefficient for the dummy capturing whether cattle are milked twice per day. We expected fewer milkings per day to be associated with better recall quality, but in fact it seems to be associated with greater measurement error.

The negative coefficient on the supplementation variable and positive coefficient on the duration of lactation variables, on the other hand, are expected, but only statistically significant for the lactation curve methods. We explain the former as reflecting greater managerial ability or simply greater importance given to animal management, and the latter to be related to the fact that the longer the milking period, the greater the degree of approximation implicit in the estimate of off-take employing the lactation curve method, and the related formula.

We found little or no impact for the other household characteristics, which is not surprising given the relative homogeneity in the socio-economic composition of the villages studied. There are two more factors which we would have wanted to control for, namely the educational level and the gender of household head. Unfortunately, the level of education of the population in the district of Dantiandou is extremely low, even by Nigerien standards, and virtually our entire sample of households is headed by a man. In other settings these variables may however play a role. We take comfort in the result that measurement error does not appear to be influenced by the enumerator collecting the data.

Finally, it is interesting to note that the overall fit of the lactation curve models is much higher compared to the other recall methods, whereas the simple 6-month recall has the lowest (with an adjusted *R*^2^ equal to zero). This suggests that the lactation curve methods likely embed a larger degree of systematic error which correlates with several variables of interest related to livestock management, which is hardly a desirable feature when employing a productivity measurement in analytical work.

## Conclusions

While there has been a renewed interested in the research over the nexus between agriculture, poverty and nutrition in recent years, associated with the increase in international food prices, this has not been matched by an improvement in the state of agricultural statistics. In Africa the availability and quality of agricultural sector data leave much to be desired, and that is particularly so for the livestock sub-sector. In terms of methods, livestock statistics offer peculiar challenges that are exemplified by the difficulties of collecting accurate milk off-take data at the household level. However, of the limited investments in livestock statistics, hardly any goes into methodological validation. The work documented in this paper takes its motivation from this state of affairs, and from the belief that given the abysmally low level of attention to this type of work, efforts to improve data quality can have substantial marginal returns and multiplier effects on research and policy analysis.

There are some clear lessons learned from work implemented in Niger to test different recall methods to capture household level milk off-take data, against a gold-standard of physical monitoring over a 12-month period.

The first is that even though there is a substantial amount of measurement error in the way even the best recall methods we tested perform in capturing household milk off-take, some methods do in fact perform fairly accurately, and much more accurately than what we expected when we designed this exercise. In particular, the methods are reliable in estimating the more common central tendency measures (mean and median), as well as the distribution of milk off-take across sample households.

The methods that rank consistently better among those we compared are the 6-month AMD recall, and a 12-month AMD recall coupled with a lactation curve recall aid. The lactation curve method, on the other hand was consistently the worst performer, with differing patterns depending on the number of data points used to estimate the off-take level at different points in the lactation. Within the AMD method, the shorter recall period appears to significantly improve the estimates, even though it is uncertain to which extent this result would hold if the 6-month recall interview were to be moved to another point in time, given the seasonality of milk off-take.

The AMD method is however more likely to return some extreme values (one of the shortcomings of this method), but this occurrence was rather limited in our sample and not frequent or large enough to undermine the overall performance of the method. In particular, not only did the AMD methods yield more accurate estimates of average milk off-take in our sample, but they also provided a more accurate depiction of the ‘true’ distribution, which is as important when assessing the role of milk (and livestock) in general in livelihoods and attempting to capture the heterogeneity across households.

Another reason militating against the use of the LC method and in favor of the AMD, is that the former seems to not only lead to larger measurement error, but also to a greater likelihood of measurement error being correlated to other variables of interest, such as herd size and length of the milking period, and hence of total milk off-take itself.

Last but not least, the LC method is in some ways more demanding on the respondent (who is prompted a few more questions) as well as the analyst, who needs to derive milk off-take estimates from the calculation of the area under the milk off-take curve. To achieve the same result, the AMD method requires fewer questions, and a much simpler multiplication of daily average off-take times the length of the off-take period. On the other hand, it should be noted that we employed a variant of the LC method that implicitly asks the respondent to ‘average out’ off-take values across all the animals milked, whereas this method is often employed with reference to specific animals within a herd. While there are reasons to discard that approach in large scale national surveys with complex questionnaires, it should also be noted that this is a limitation of the results presented here, which do not provide a comparative evaluation of the LC method when implemented with reference to specific animals.

Another limitation of the study concerns its external validity, that is, the extent to which the conclusions apply to survey data collection in other areas in Sub-Saharan Africa or in other developing regions, and to animals other than dairy cattle. Both concerns can only be addressed by replicating similar methodological validation exercises in different settings. Ancillary evidence to the results presented in the paper point to the fact that the distributions of milk off-take estimates may perform very differently for large and small ruminants, due to the shorter lactation periods of the latter.

Taken together, the results presented in this paper have clear implications for future questionnaire design that we feel are strong enough to recommend using the better performing methods in future household surveys on small livestock keepers in extensive livestock systems in low-income settings, at least when the menu of workable options is one that includes the alternative we have tested. While there are limits to the external validity of these results, which should be repeated in different settings and for different species, we do maintain that the findings reported here are strong enough to be already taken up in future questionnaire design by National Statistical Offices, researchers, and anyone involved in household survey design.

## Figures and Tables

**Table 1:** Summary statistics for different randomly selected sub-samples.

		Avg. raised cows	Avg. lactating cows	Milking months	Avg. cows milked	Length previous lactation	Gap in last two births	Age cow at first birth	Number of births	Age of cow	
Unit of measurement		units	units	months	units	months	months	months	units	years	
Type of questionnaire											
Lactation Curve Quest.	*n*	172	170	168	168	172	155	168	169	168	*Full sample*
	*Mean*	5.6	2.7	11.1⁎	2.0	12.4	22.2	52.2	3.0	10.6
	*Median*	4	2	12	2	12	24	60	3	10
	*Std. Dev.*	5.1	1.8	2.3	1.2	4.4	5.3	24.5	1.4	3.1
	*Min*	1	1	2	1	4	12	4	1	5
	*Max*	31	10	12	7	30	36	108	7	19

Avg/Herd/Day (AMHD) Quest.	*n*	168	164	163	163	157	154	164	165	164
*Mean*	5.9	2.8	10.5⁎	2.0	12.6	22.3	52.2	3.0	10.3
*Median*	4	2	12	2	12	24	60	3	10
*Std. Dev.*	5.5	2.1	3.0	1.2	4.9	5.4	24.0	1.5	3.0
*Min*	1	1	1	1	3	12	3	1	5
*Max*	35	12	12	7	28	36	96	9	18

Lactation Curve Quest.	*n*	135	135	135	135	135	122	133	134	133	*Monitored*
*Mean*	5.9	2.8	11.1	2.0	12.5	22.1	51.6	3.1	10.5
*Median*	5	2	12	2	12	24	60	3	10
*Std. Dev.*	5.2	1.9	2.2	1.1	4.4	5.3	24.4	1.4	3.0
*Min*	1	1	2	1	5	12	4	1	5
*Max*	31	10	12	6	30	36	96	7	19

Avg/Herd/Day (AMHD) Quest.	*n*	134	134	134	134	127	125	134	134	134
*Mean*	6.2	2.9	10.7	2.1	12.3	22.0	51.8	3.0	10.4
*Median*	5	2	12	2	12	23	60	3	10
*Std. Dev.*	5.8	2.2	2.8	1.2	4.6	5.4	24.3	1.5	3.1
*Min*	1	1	1	1	3	12	4	1	6
*Max*	35	12	12	7	28	36	96	9	18

Lactation Curve Quest.	*n*	37	35	33	33	37	33	35	35	35	*Not monitored*
*Mean*	4.6	2.4	10.8	2.0	11.9	22.4	54.6	3.0	10.9
*Median*	3	2	12	2	12	22	60	2	10
*Std. Dev.*	4.7	1.6	2.7	1.5	4.4	5.5	24.7	1.5	3.6
*Min*	1	1	2	1	4	12	5	1	5
*Max*	25	7	12	7	24	36	108	7	17

Avg/Herd/Day (AMHD) Quest.	*n*	34	30	29	29	30	29	30	31	30
*Mean*	4.4	2.1	9.6	1.9	13.8	23.8	54.3	2.8	9.8
*Median*	3	2	12	2	12	24	60	2	10
*Std. Dev.*	3.8	1.4	3.6	1.2	5.7	5.4	22.6	1.3	3.0
*Min*	1	1	1	1	4	12	3	1	5
*Max*	14	5	12	5	24	36	96	7	17

Note: The three panels in the table refer to the entire sample, the subset of households whose production was physically monitored, and the subset that only received the recall questionnaire. See text for more explanation.

⁎ Significance level: 10%.

**Table 2:** Household milk off-take (liters). Comparison of monitoring and recall data, various methods (annual and 6-month).

	All households						Village households						Camp households					
	n	Mean	Median	Std. Dev.	Min	Max	n	Mean	Median	Std. Dev.	Min	Max	n	Mean	Median	Std. Dev.	Min	Max
*Physical monitoring*																		
Monitoring during 12 months	300	877	741	631	10	3291	129	605	512	465	10	2484	171	1083	971	662	45	3291

*Recall on 12 months*																		
6-month recall – annualized	171	847	720	699	8	3600	63	569	360	534	8	3240	78	1089	1080	640	180	2880
Avg./Herd/Day (AMHD) – LC module	167	934	720	870	43	5400	55	684	557	591	43	2229	79	1072	929	845	130	4458
Avg./Herd/Day (AMHD) – All	330	926	720	863	9	6687	111	759	557	692	9	3960	157	1049	743	880	130	6687
Avg./Herd/Day (AMHD) – 12 months recall	163	918	720	859	9	6687	56	832	549	777	9	3960	78	1027	743	920	130	6687
Lactation curve – 3 points	167	1200	818	1146	132	6900	56	1091	600	1140	132	6037	79	1284	913	1229	201	6900
Lactation curve – 4 points	167	990	693	934	87	6037	56	915	480	1010	87	6037	79	1055	855	954	174	5263

Note: 330 sample of households with milk production																		

Monitoring during 6 months	300	471	386	323	10	1825	129	334	267	230	10	1321	171	574	509	345	45	1825
Recall after 6 months	171	424	360	350	4	1800	63	284	180	267	4	1620	78	545	540	320	90	1440

Note: 300 monitored households (152 with LC quest./148 with AMHD quest.)																		

**Table 3:** Correlation and regression (Ordinary Least Squares, OLS) coefficients between monitoring and recall methods.

	Correlation coefficient	OLS no constant		OLS		OLS (logs)		*n*
	Coeff	*R*^2^	Coeff	*R*^2^	Coeff	*R*^2^
*Correlation with 12 months monitoring*								
6-month recall – annualized	0.71	0.91	0.81	0.68	0.50	0.76	0.63	141
Avg./Herd/Day (AMHD) – LC module	0.61	0.79	0.72	0.51	0.38	0.57	0.48	134
Avg./Herd/Day (AMHD) – All	0.52	0.73	0.66	0.41	0.27	0.58	0.44	268
Avg./Herd/Day (AMHD) – 12-month recall	0.44	0.69	0.60	0.33	0.19	0.58	0.41	134
Lactation curve – 3 points	0.35	0.47	0.52	0.19	0.12	0.47	0.21	135
Lactation curve – 4 points	0.36	0.57	0.53	0.24	0.13	0.49	0.24	135

*Correlation with 6 months monitoring*								
Recall after 6 months	0.67	0.97	0.78	0.69	0.44	0.76	0.63	141

**Table 4:** Regressions’ results on the determinants of the measurement errors.

	(1)	(2)	(3)	(4)	(5)	(6)	(7)
	6-month recall annualized	Avg/Herd/Day LC module	Avg/Herd/Day All	Avg/ Herd/Day 12-month recall	Lactation Curve 3-points	Lactation Curve 4-points	Recall after 6 months
Coefficient of variation, all cows	−0.514 (0.317)	−0.127 (0.551)	0.468 (1.075)	0.463 (1.698)	−0.986 (1.148)	−0.527 (0.960)	−0.253 (0.230)
Dummy Territory (1 = Camp 0 = Village)	0.121 (0.229)	−0.404⁎ (0.209)	−0.393 (0.323)	−0.233 (0.486)	−0.780⁎⁎ (0.339)	−0.618⁎⁎ (0.279)	0.252 (0.182)
Dummy if milk collected only in the morning	0.290 (0.186)	0.367⁎ (0.195)	0.680⁎⁎ (0.303)	0.974⁎ (0.574)	0.430 (0.323)	0.405 (0.290)	0.250⁎ (0.137)
Dummy collector	−0.119 (0.186)	−0.016 (0.173)	−0.036 (0.234)	−0.108 (0.473)	0.073 (0.376)	−0.107 (0.318)	−0.073 (0.135)
Log of number of cows	−0.106 (0.142)	0.677⁎⁎⁎ (0.190)	0.783⁎⁎⁎ (0.204)	0.962⁎⁎⁎ (0.327)	1.232⁎⁎⁎ (0.380)	1.143⁎⁎⁎ (0.371)	−0.033 (0.120)
Number of supplemented cows	−0.052 (0.039)	−0.040 (0.050)	−0.046 (0.033)	−0.068 (0.053)	−0.258⁎⁎⁎ (0.079)	−0.222⁎⁎⁎ (0.069)	−0.003 (0.032)
Log of age of hh head	−0.095 (0.277)	0.510⁎⁎ (0.242)	0.235 (0.355)	0.126 (0.664)	0.405 (0.514)	0.423 (0.449)	0.141 (0.180)
Annual revenues of the exodus of family members in 1000 FCFA	0.000 (0.000)	−0.000 (0.000)	−0.000 (0.000)	−0.000 (0.001)	−0.000 (0.000)	−0.000 (0.000)	0.000 (0.000)
Dummy if agriculture is the only hh activity	0.028 (0.151)	−0.391⁎ (0.229)	−0.370⁎ (0.201)	−0.410 (0.347)	0.013 (0.339)	0.048 (0.310)	0.036 (0.126)
Number of mobile phones owned in the hh	0.102 (0.063)	0.040 (0.098)	−0.027 (0.077)	−0.192 (0.157)	0.151 (0.195)	0.182 (0.198)	0.060 (0.056)
Number of other animals (camels, donkeys)	0.051 (0.047)	0.021 (0.051)	−0.050 (0.053)	−0.165 (0.112)	0.018 (0.069)	−0.003 (0.062)	0.023 (0.039)
Duration of previous lactation (months)	0.002 (0.015)	−0.017 (0.024)	−0.041 (0.028)	−0.051 (0.041)	0.175⁎⁎⁎ (0.060)	0.142⁎⁎ (0.057)	−0.006 (0.010)
Constant	0.511 (1.068)	−1.523 (1.187)	−0.232 (1.384)	0.726 (2.225)	−2.418 (2.485)	−2.711 (2.386)	−0.743 (0.689)

Observations	129	134	266	132	135	135	129
Log-likelihood	−146.6	−181.9	−533.9	−299.1	−254.8	−240.2	−115.2
Prob. > *F*	0.773	0.000	0.001	0.124	0.000	0.000	0.413
*R*-squared	0.105	0.199	0.091	0.100	0.370	0.340	0.094
Adj. *R*-squared	0.013	0.120	0.048	0.009	0.308	0.275	−0.000
RMSE	0.795	0.990	1.846	2.457	1.681	1.508	0.623

Note: Robust standard errors in parentheses.

⁎ *p* < 0.10.

⁎⁎ *p* < 0.05.

⁎⁎⁎ *p* < 0.01.

## Footnotes

1 For information on the two projects see http://www.fao.org/ag/againfo/programmes/en/Livestock_Africa_Data_Policies.html and www.worldbank.org/lsms.

2 In fact, there are two variants of the AMD method, one that asks about Average Milk *per Animal* per Day (AMAD), and one that asks about Average Milk *per Herd* per Day (AMHD). Our results refer mainly to the latter even though – as we explain in what follows – we used both at different points in our fieldwork.

3 An alternative way of computing milk production is to assume a monotonic decline in lactation from a peak after a week from parturition, to zero at the end of the lactation (milk production) period.

4 One source of measurement error we do not focus on here is the tendency of respondents to recall question to report figures in round numbers (e.g. quarter of a liter or half a liter), whereas for physical monitoring measures were recorded in actual cubic centimeters. This is a well-known phenomenon (see Roberts and Brewer, 2001). For an application to land size measures in agricultural surveys see Carletto et al. (2013).

5 Note that the area of the rectangle ABCD depends on the mean milk off-take per animal and day (BC) but also of the mean duration of milking (AB). The latter is much more variable and depends on factors such as individual, parity, season, and it could reflect a progressive reduction of milking frequency.

6 Final report and documents available at http://eclis.get.obs-mip.fr/.

7 One possible reason is that the 6-month recall survey occurred at the end of the wet season, so that the average milk off-take was mostly based on the high milk off-take during the wet season while the 12-month survey occurred at the end of the dry season, requiring of the herder a more difficult averaging exercise between low milk off-take in the last months and higher off-take in the former months.

8 In addition, a smaller scale pilot survey that was run in February 2011 on about 60 households provided qualitative confirmation of the expectation that the standard LSMS module tends to understate milk off-take compared to other recall methods.

9 Data not reported, but available from the authors upon request.

10 The annualized 6-month recall is just the 6-month recall times 2. What changes in the comparison between the two is the benchmark data, which is the full 12 months of monitoring in the former case, and the first 6 months of monitoring in the latter.

11 It should be noted that while we treat milk monitoring as the benchmark this measure is also, as any measure, affected by some degree of error.
